# From Bench Testing to Virtual Implantation: A Comparative Study Between Poly‐l‐Lactic Acid and Nickel‐Titanium Braided Stents

**DOI:** 10.1002/cnm.70078

**Published:** 2025-08-07

**Authors:** Agnese Lucchetti, Levi G. Juhl, Anna Corti, Alissa Zaccaria, Thomas Gries, Claudio Chiastra, Ted J. Vaughan, Dario Carbonaro

**Affiliations:** ^1^ Institut für Textiltechnik of RWTH Aachen University Aachen Germany; ^2^ Department of Electronics Information and Bioengineering Milan Italy; ^3^ Consorzio Intellimech Bergamo Italy; ^4^ PolitoBIOMed Lab, Department of Mechanical and Aerospace Engineering, Politecnico di Torino Turin Italy; ^5^ Biomechanics Research Centre (BioMEC), School of Engineering and Institute for Health Discovery and Innovation, College of Science and Engineering University of Galway Galway Ireland

**Keywords:** bioresorbable stent, finite element analysis, peripheral artery disease, polymeric stent

## Abstract

Bioresorbable braided stents represent a promising solution for the treatment of peripheral artery disease, providing temporary mechanical support before gradually degrading into biocompatible byproducts. Previous studies have highlighted their lower mechanical performance compared to permanent metallic stents. However, their implantation in lower limb arteries remains unexplored, leaving uncertainty on whether their mechanical performance is sufficient for effective treatment. The aim of the present study was to evaluate the performance of a poly‐l‐lactic acid (PLLA) braided stent for the treatment of lower limb arteries through in silico analysis and compare it with that of a nickel‐titanium (NiTi) device. A finite element (FE) model of the PLLA stent was implemented and validated against experimental bench test data. Subsequently, the mechanical characteristics of the PLLA device were compared to those of a NiTi stent, with identical geometrical features, through FE simulations of two bench tests (i.e., parallel plate compression and crimping tests). Finally, a virtual implantation procedure of both devices in a patient‐specific lower limb artery was conducted by FE analysis, accounting for three different arterial wall conditions, to compare the stents' treatment performance. The FE analysis of the bench tests confirmed that the PLLA stent generated much lower force magnitudes than the NiTi device. Moreover, the virtual implantation procedure indicated the limited short‐term performance of the PLLA stent for the treatment of peripheral artery disease in terms of risk for permanent deformations, low lumen gain, high values of incomplete stent apposition and a nonuniform distribution of contact pressure on the arterial wall.

## Graphical abstract

A computational procedure was developed to investigate the mechanical characteristics and implantation performance of a bioresorbable braided stent for the treatment of lower limb arteries and to compare it with a permanent metallic device. In silico bench tests showed that the bioresorbable stent generated substantially lower force magnitudes compared to the permanent metallic device. Implantation simulations revealed the bioresorbable stent's limited short‐term treatment performance, with an associated risk of permanent deformations, low lumen gain and stent malapposition.

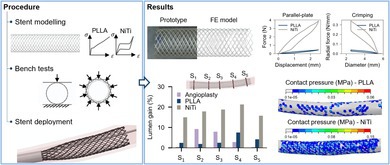

**Abbreviations**
COF: chronic outward force
FE: finite element
ISA: incomplete stent apposition
LG: lumen gain
MLA: minimum lumen area
NiTi: nickel–titanium
PAD: peripheral artery disease
PLLA: poly‐l‐lactic acid
RIF: radial interaction force
RRF: radial resistive force
SFA: superficial femoral artery

## 1 Introduction

Braided stents consist of intertwined wires, forming a mesh‐like tubular structure, with individual wires capable of relative motion [1]. These devices are implanted through a minimally invasive procedure, during which the stent is positioned at the arterial lesion and, upon release from the catheter, it self‐expands to provide structural support to the arterial wall, thereby preventing vessel elastic recoil and reocclusion postintervention [2]. One of the key advantages of braided stents is their ability to conform to the vessel's shape and motion, making them particularly suitable for treating peripheral artery disease (PAD) in the lower limb arteries, such as the superficial femoral artery (SFA), which undergo extensive movement [3, 4]. Furthermore, braided devices offer tunable design parameters, including braiding angle, wire diameter, number of wires and braiding pattern, allowing for customization of the mechanical characteristics to meet specific clinical needs [5, 6]. Braided stents can be manufactured from various materials, including metals and polymers, either permanent or bioresorbable. Commercially available devices, widely adopted for the treatment of PAD in the SFA, are mainly fabricated from wires made of permanent metals such as nickel–titanium (NiTi) or cobalt–chromium–nickel [7, 2, 8, 9]. Conversely, bioresorbable braided stents, made purely from polymers such as poly‐l‐lactic acid (PLLA), are currently under development. These stents offer the advantage over permanent metallic stents of providing temporary vessel support for the required duration before gradually degrading into biocompatible byproducts [10]. This feature confers several long‐term benefits, including the prevention of late inflammation, reduced imaging artifacts in clinical images, reduction of complications during secondary surgeries and a decreased need for long‐term antiplatelet therapy [10, 11, 12].

Several studies have been performed to investigate the mechanical characteristics of bioresorbable braided stents, with the goal of evaluating their clinical efficacy. Lucchetti et al. [13] conducted experimental bench tests to investigate the mechanical performance of PLLA devices by considering various design parameters and loading scenarios. Furthermore, the degradation behaviour of the same devices was investigated in vitro both in real‐time and thermally accelerated conditions [14]. Zhao et al. [15] analysed different computational modelling techniques of PLLA braided stents, while Carbonaro et al. [16] carried out a computational optimization study aimed at enhancing the mechanical properties of these devices. Finally, Li et al. [17] conducted a combined experimental and computational study to analyse the deformation behaviour of PLLA wires, assessing the risk of permanent deformations that could compromise the stent's mechanical performance. These studies focused on mechanical characterisation through bench testing and confirmed the inferior mechanical performance of bioresorbable stents compared to their metallic counterparts, attributable to the lower mechanical properties of bioresorbable polymers relative to metals. In this setting, however, the implantation procedure of bioresorbable braided devices in SFAs has not yet been investigated, through either in silico or in vitro models, leaving it unclear whether their mechanical properties are adequate for effective treatment.

In this context, the aim of the present study was to evaluate and compare the performance of PLLA and NiTi braided devices for the treatment of lower limb arteries through in silico analysis. First, a finite element (FE) model of a bioresorbable braided stent made of PLLA was implemented and validated against experimental bench test data. Subsequently, the mechanical characteristics of the stent were compared to those of a NiTi device through FE simulations of two different bench tests, namely parallel plate compression and crimping tests. Finally, the implantation procedure into a patient‐specific SFA was virtually simulated using FE analysis, accounting for different arterial wall conditions.

## 2 Methods

**FIGURE 1:**
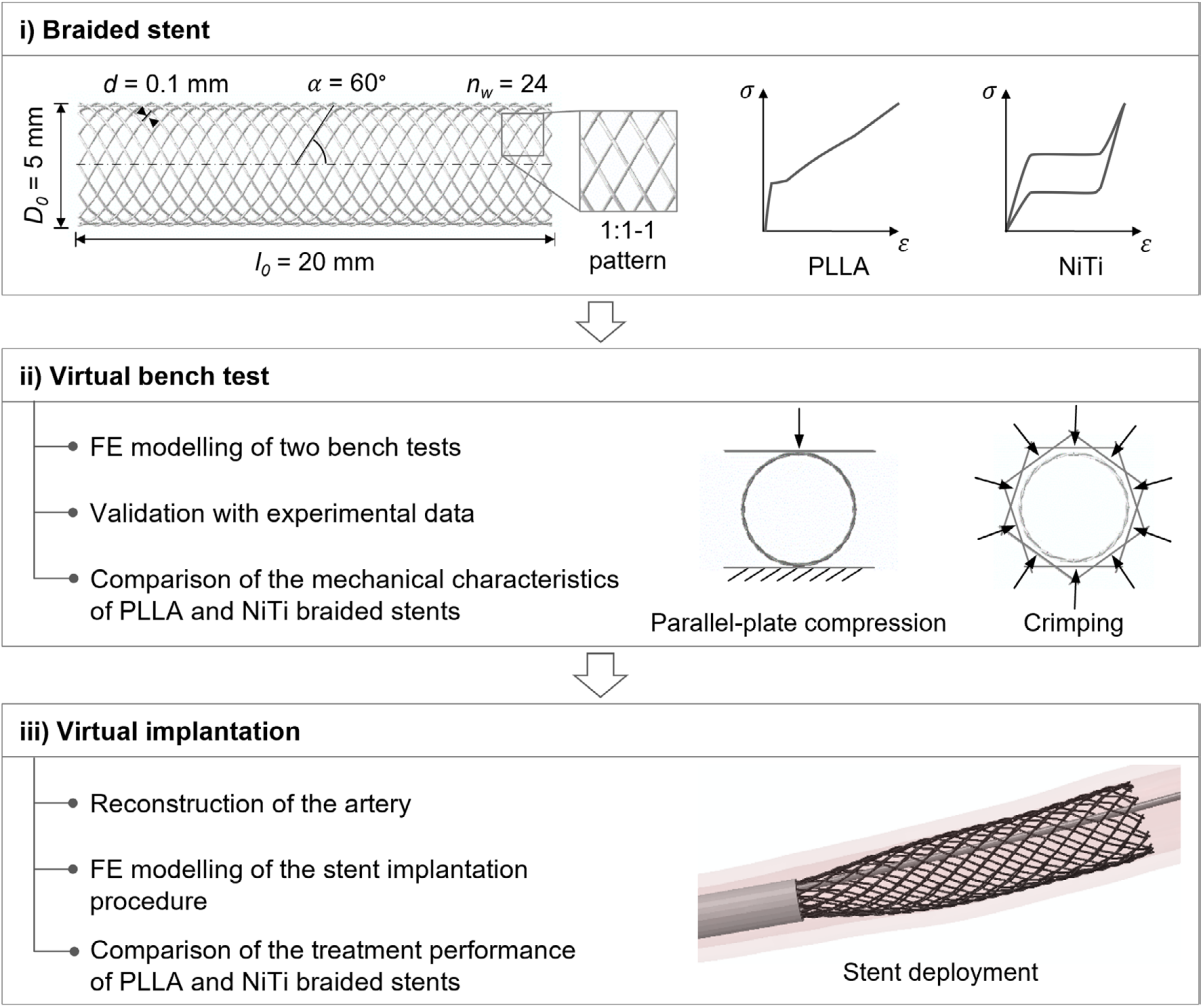
Main steps of the present study: (i) FE modelling of braided stents designed for the treatment of the SFA with material properties of PLLA and NiTi; (ii) FE modelling of two bench tests to compare the mechanical characteristics of PLLA and NiTi devices and (iii) FE modelling of the implantation procedure into a patient‐specific SFA model to compare the performance of PLLA and NiTi braided stents. The stent geometrical features are illustrated in (i) and include: stent diameter (*D*_0_), stent length (*l*_0_), wire diameter (*d*), braiding angle (α), number of wires (*n*_*w*_) and one‐over‐one‐under (1:1‐1) braiding pattern.

The study followed three main steps (Figure 1): (i) FE modeling of braided stents with the material properties of PLLA and NiTi, with geometrical features designed for the treatment of the SFA; (ii) FE modeling of two bench tests for stent's mechanical characterisation, including validation of the FE models with experimental data and comparison of the mechanical characteristics of PLLA and NiTi devices; (iii) FE modeling of the stent implantation procedure and comparison of the treatment performance between PLLA and NiTi braided stents.

### 2.1 FE Model of the Braided Stent

The geometry of the braided stent was generated using parametric equations as detailed in prior studies [9, 16, 18]. Specifically, a device designed for the treatment of PAD in SFAs was considered. The geometrical features were derived from Lucchetti et al. [13] and included: internal diameter *D*_0_ = 5 mm, initial length *l*_0_ = 20 mm, wire diameter *d* = 0.1 mm, braiding angle α = 60°, number of wires *n*_*w*_ = 24 and one‐over‐one‐under (1:1‐1) pattern (Figure 1). The stent geometry was generated in Matlab (MathWorks, Natick, MA, USA) and imported in Abaqus/Explicit (Dassault Systèmes Simulia Corp., Johnston, RI, USA). The geometry was discretized using B31 Timoshenko beam elements with three elements between each crossing point, following a mesh independence study [18]. Two materials, PLLA and NiTi, were implemented in the FE model for comparison. Specifically, an elasto‐plastic constitutive model was considered for PLLA, with values of the material model parameters reported in Table 1, derived from uniaxial tensile tests on PLLA wires [13]. A super‐elastic constitutive model was adopted for NiTi [19], with values of the material model parameters provided in Table 2, based on a previous study on NiTi braided devices [4].

**TABLE 1:** Parameters of the elasto‐plastic material constitutive model for PLLA [13].

Material model parameter	Value
Young's modulus	6.4 GPa
Poisson's ratio	0.35
Yield stress	110 MPa
Ultimate tensile strength	310 MPa
Elongation at break	0.53

**TABLE 2:** Parameters of the NiTi super‐elastic material constitutive model for NiTi [4].

Material model parameter	Value
Austenite elastic modulus	56.7 GPa
Austenite Poisson's ratio	0.3
Martensite elastic modulus	25.6 GPa
Martensite Poisson's ratio	0.3
Transformation strain	0.046
Start of transformation loading	590 MPa
End of transformation loading	600 MPa
Start of transformation unloading	420 MPa
End of transformation unloading	380 MPa

### 2.2 Virtual Bench Tests

**FIGURE 2:**
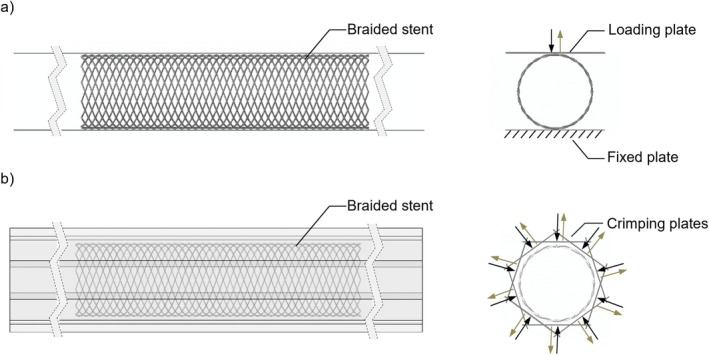
Schematic of the two bench tests: (a) parallel‐plate compression and (b) crimping. Each test consisted of a compression and an expansion step, indicated respectively with the black and brown arrows in the figure.

Parallel‐plate compression and crimping are two standard bench tests for the mechanical characterisation of vascular stents [20]. FE simulations of the two tests were conducted with Abaqus/Explicit to characterise the mechanical performance of PLLA and NiTi devices (Figure 2). The plates of the testing machines were modelled as rigid and discretized with R3D4 elements [21]. Specifically, for the parallel‐plate compression, two plates were modelled. The lower plate was fixed and the upper plate was displaced vertically downwards to compress the stent by 50% of its initial diameter and then released to its original position [13, 20] (Figure 2a). For the crimping, 10 plates were modelled, which were radially displaced to compress the stent to 50% of its initial diameter and then released to their original position [13, 20] (Figure 2b). Interactions between the parts were implemented with the general contact algorithm, considering the default hard normal contact behaviour, with tangential behaviour defined by friction coefficients. Specifically, the friction coefficient between the stent and the plates was set to *μ*_stent‐plates_ = 0.25 for parallel‐plate compression and *μ*_stent‐plates_ = 0.13 for crimping. The coefficient for self‐contact among the stent wires was set to *μ*_stent‐self_ = 0.3 for both tests. These values were determined following a calibration procedure, details of which can be found in Lucchetti et al. [14]. Within the simulation, it was ensured that the ratio between kinetic and internal energy was less than 5% to perform a quasi‐static analysis [22]. The primary outputs of the parallel plate compression and radial tests were the force and radial force generated by the device, respectively. The radial force was quantified as the sum of the forces' magnitude exerted on the moving plates. Forces measured in the parallel‐plate compression and crimping were divided by the stent initial length (*l*_0_).

The FE model of the PLLA braided stent was validated using experimental data from Lucchetti et al. [13]. First, the FE model was visually compared with the available prototype of the PLLA device. Subsequently, to verify the accuracy of the calibrated friction coefficients, a denser mesh stent (α = 70°) was simulated and the results were compared with the experimental curve reported by Lucchetti et al. [13]. The FE models of the two bench tests were then utilised to characterise and compare the mechanical performance of the PLLA and NiTi devices featuring α = 60°.

### 2.3 Virtual Stent Implantation

FE analyses of stent implantation in a patient‐specific diseased SFA were conducted to assess the devices' treatment performance. In line with clinical practice, angioplasty was simulated in the lesion segment of the artery before the implantation procedure [23, 24]. The FE model of stent implantation comprises the following main components in addition to the stent: (i) artery, (ii) balloon and (iii) delivery system.

#### 2.3.1 FE Models

##### 2.3.1.1 Artery

**FIGURE 3:**
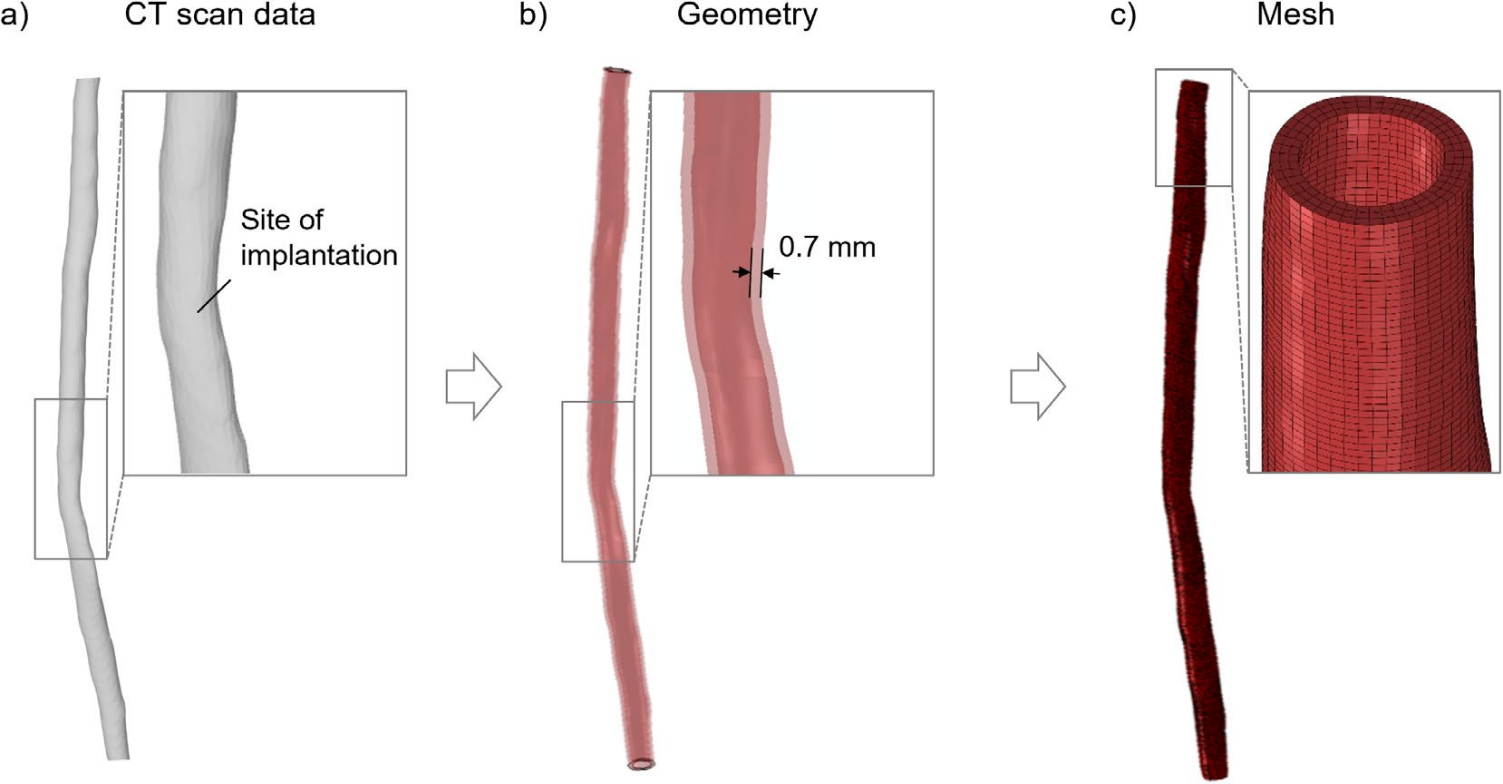
Procedure for the implementation of FE model of the SFA: (a) reconstructed CT scan of the SFA and identification of the site of implantation, (b) SFA geometry and (c) SFA mesh.

The inner surface of the SFA of a patient affected by severe PAD reconstructed from computed tomography was made available by Boston Scientific Ltd. Co. (Galway, Ireland) through the BioImplant ITN project funded by the European Union's Horizon 2020 research and innovation programme (grant agreement no 813869) (Figure 3a). The region of the SFA with a minimum inner diameter of 2.7 mm was identified as the site of treatment (Figure 3a). The geometry of the artery was generated from the inner surface, assuming a constant vessel wall thickness of 0.7 mm [25] (Figure 3b). The geometry was meshed with C3D8R hexahedral elements, considering three layers of elements along the vessel wall (Figure 3c) [26]. The material of the artery was assumed to be homogeneous and isotropic, following the modelling approach proposed by Noble et al. [27], who conducted mechanical inflation tests on 10 ex vivo pathological femoral arteries and proposed a Neo‐Hookean incompressible hyper‐elastic constitutive material model to homogenise the arterial tissue. Accordingly, in the present study, three values of material model parameters from Noble et al. [27] were considered to represent varying tissue stiffness, corresponding to different arterial wall conditions (Table 3). Moreover, perfect plasticity with yield stress of 0.14 MPa was introduced to model plaque fracture, based on experimental tensile tests on calcified femoral plaques [28], as it was assumed that after yielding the material has no resistance.

**TABLE 3:** Parameter of the incompressible Neo‐Hookean hyper‐elastic material constitutive model used for the SFA model.

Material model parameter value	Arterial wall condition
40.87 kPa	Low stiffness
92.09 kPa	Medium stiffness
143.80 kPa	High stiffness

*Note:* The three values correspond to the minimum, medium and maximum values reported by Noble et al. [27].

##### 2.3.1.2 Balloon

The FE model of the balloon adopted for the angioplasty was implemented following the approach proposed by Corti et al. [29]. The Armada 35 Balloon Dilatation Catheter (Abbott, Illinois, USA) was considered, which is commonly adopted for angioplasty in lower limb arteries. Details on the balloon FE model are available in the Supporting Information.

###### 2.3.1.2.1 Delivery System

**FIGURE 4:**
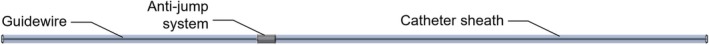
Delivery system including the guidewire, the catheter sheath and the anti‐jump system.

The FE model of the delivery system was based on the model by Zaccaria et al. [22] and comprised a NiTi guidewire, a 6‐Fr Polytetrafluoroethylene catheter sheath and an anti‐jump system (Figure 4). Further details are provided in the Supporting Information.

#### 2.3.2 FE Simulation of the Implantation Procedure

**FIGURE 5:**
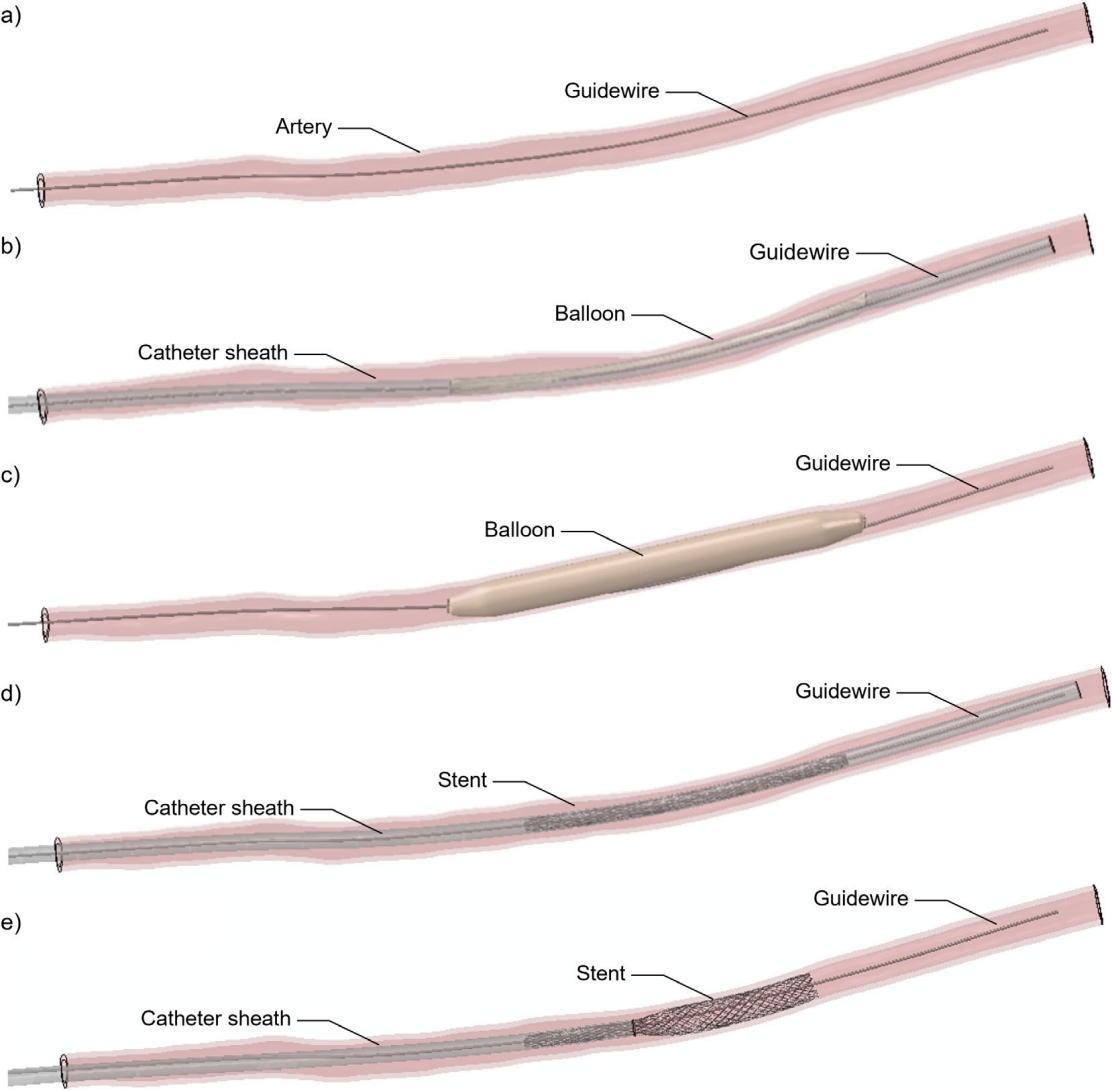
Main steps of the FE simulation of the implantation procedure: (a) guidewire insertion, (b) morphing of the balloon delivery system, (c) angioplasty, (d) morphing of the stent delivery system and (e) stent implantation.

The FE simulation of the stent implantation procedure comprises five main steps, following the approach proposed by Zaccaria et al. [22] (Figure 5): (i) guidewire insertion; (ii) morphing of balloon delivery system; (iii) angioplasty; (iv) morphing of stent delivery system and (v) stent implantation. Specifically:

- The guidewire was inserted by displacing the node at the proximal extremity of the guidewire while activating its contact with the artery (Figure 5a). To prevent interpenetration between the catheter and artery in the following steps, the guidewire contact diameter was artificially increased to a value greater that the catheter sheath diameter. The two extremities of the artery were fixed in all degrees of freedom in all five steps of the implantation procedure. Moreover, pre‐loading on the artery due to physiological internal pressure was not considered.
- The balloon delivery system was inserted through a morphing procedure (Figure 5b), using a morphing wire discretized with the same number of nodes as the guidewire. A displacement was applied to each node of the morphing wire to match the shape of the guidewire within the artery, thereby deforming the catheter sheath and balloon.
- The angioplasty was divided into three steps: inflation, deflation and stabilization. Inflation was performed by applying a pressure of 2.1 MPa to the balloon inner surface (Figure 5c). The pressure was then released in the deflation step, followed by a stabilisation step to complete the procedure. The pressure value applied was selected based on the manufacturer's compliance chart.
- The stent delivery system was inserted, repeating the morphing procedure adopted for the balloon delivery system (Figure 5d).
- The braided stent was implanted by displacing nodes at the catheter sheath's distal extremity to retract the catheter sheath, thus allowing the device free expansion (Figure 5e).

The FE simulation was carried out in Abaqus/Explicit as a quasi‐static process, ensuring that the ratio between kinetic and internal energy was less than 5% to perform a quasi‐static analysis [22]. Interactions between the parts were implemented with the general contact algorithm, considering the default hard normal contact behaviour, with tangential behaviour defined by friction coefficients. Specifically, the friction coefficient between the stent and the delivery system (i.e., catheter sheath and guidewire) with the vessel was set to *μ*_stent‐vessel_ = 0.05, the friction coefficient for self‐contact among the stent wires was set to *μ*_stent‐self_ = 0.3, while the contact between the stent and the catheter during stent deployment was defined as frictionless.

#### 2.3.3 Verification of the FE Simulation

To verify the credibility of the FE analysis of the stent implantation, the radial interaction force (RIF) magnitude exerted by the implanted stent to the artery was measured and compared to the chronic outward force (COF) obtained from the crimping test at the artery diameter. Specifically, the magnitude of the RIF was computed as the sum of the magnitudes of the normal contact forces of all the nodes of the stent.

#### 2.3.4 Evaluation of the Treatment Performance

**FIGURE 6:**
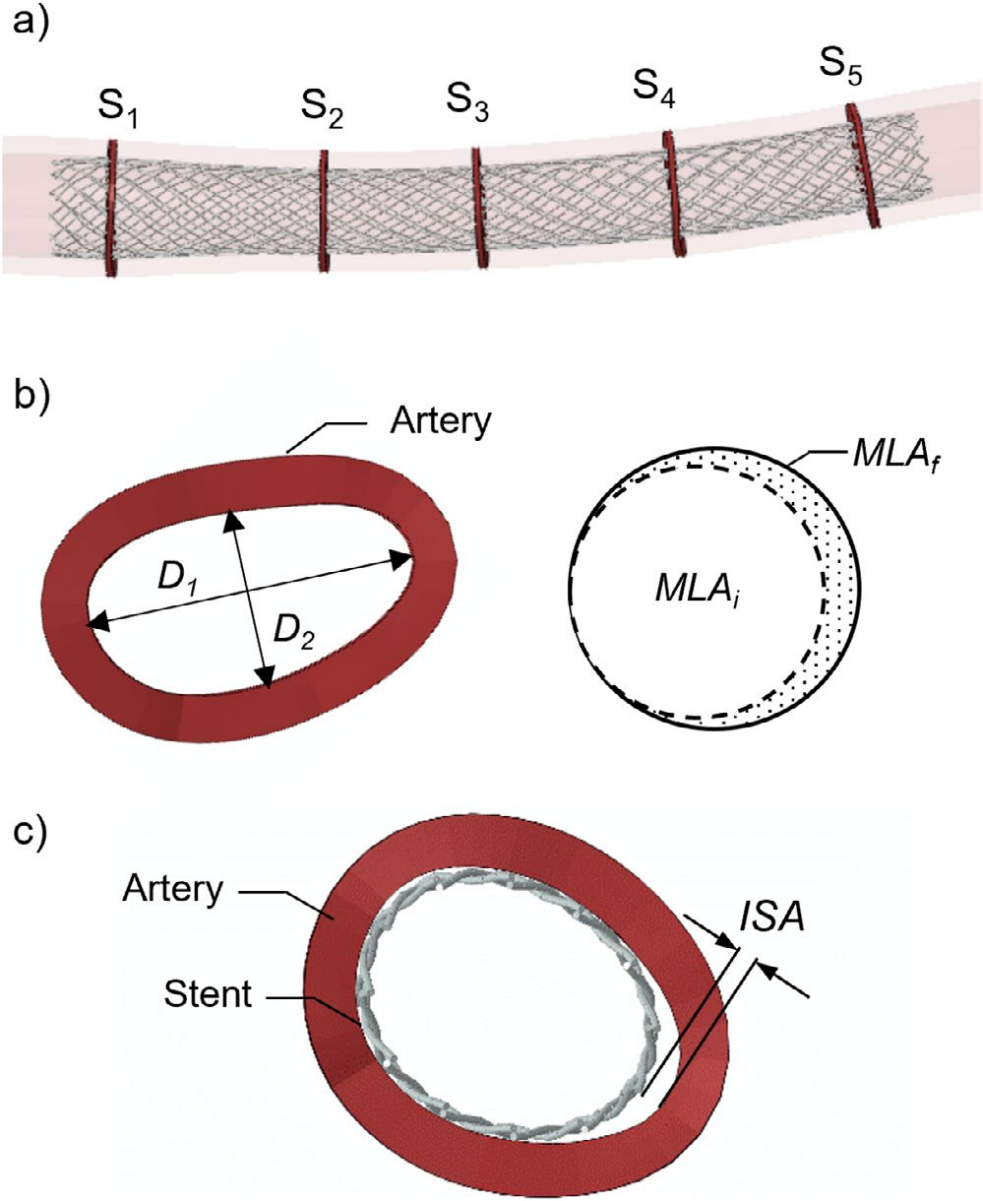
(a) Cross‐sections *S*_1_–*S*_5_ for the evaluation of the treatment performance; (b) distances *D*_1_ and *D*_2_ for the evaluation of the MLA according to Equation (1). LG measured as relative difference of MLA at the beginning (i.e., MLA_*i*_) and end (i.e., MLA_*f*_) of each step according to Equation (2) and (c) ISA measured as the maximum distance between the braided stent and the artery internal wall.

The following quantities were quantified to assess the treatment performance of the braided stents: (i) the maximum principal stress of the device when inserted into the catheter sheath, (ii) the lumen gain (LG) induced by both the angioplasty and the stent implantation, (iii) the incomplete stent apposition (ISA) and (iv) the contact pressure on the arterial wall following stent deployment. Specifically, the LG was measured at five cross‐sections distributed along the lesion of the artery (Figure 6a, *S*_1_–*S*_5_) both post‐angioplasty and post‐stent deployment. At each cross‐section, two distances, *D*_1_ and *D*_2_, were quantified before and after the treatment to compute the minimum lumen area (MLA) (Figure 6b):

(1) $$\mathrm{MLA}=\frac{1}{4}\cdot \pi \cdot D_{1}\cdot D_{2}$$

Accordingly, the LG was calculated as follows:

(2) $$\mathrm{LG}=\frac{\mathrm{ML}A_{f}-\mathrm{ML}A_{i}}{\mathrm{ML}A_{i}}$$

where MLA_*i*_ and MLA_*f*_ represent the MLA at the beginning and end of the step, respectively. Specifically, the LG induced by stent implantation was computed using the post‐angioplasty artery configuration as the initial state. The ISA was measured as the maximum distance between the stent struts and the artery internal wall (Figure 6c). Specifically, the ISA was assumed to be critical for values higher than 0.1 mm, corresponding to the wire diameter *d* of the stent, as values greater than *d* could promote stent thrombosis, by disturbing the normal laminar flow along the vessel wall, as well as reduce re‐endothelialisation and neointima formation [30].

## 3 Results

### 3.1 FE Model of the Stent and Virtual Bench Tests

**FIGURE 7:**
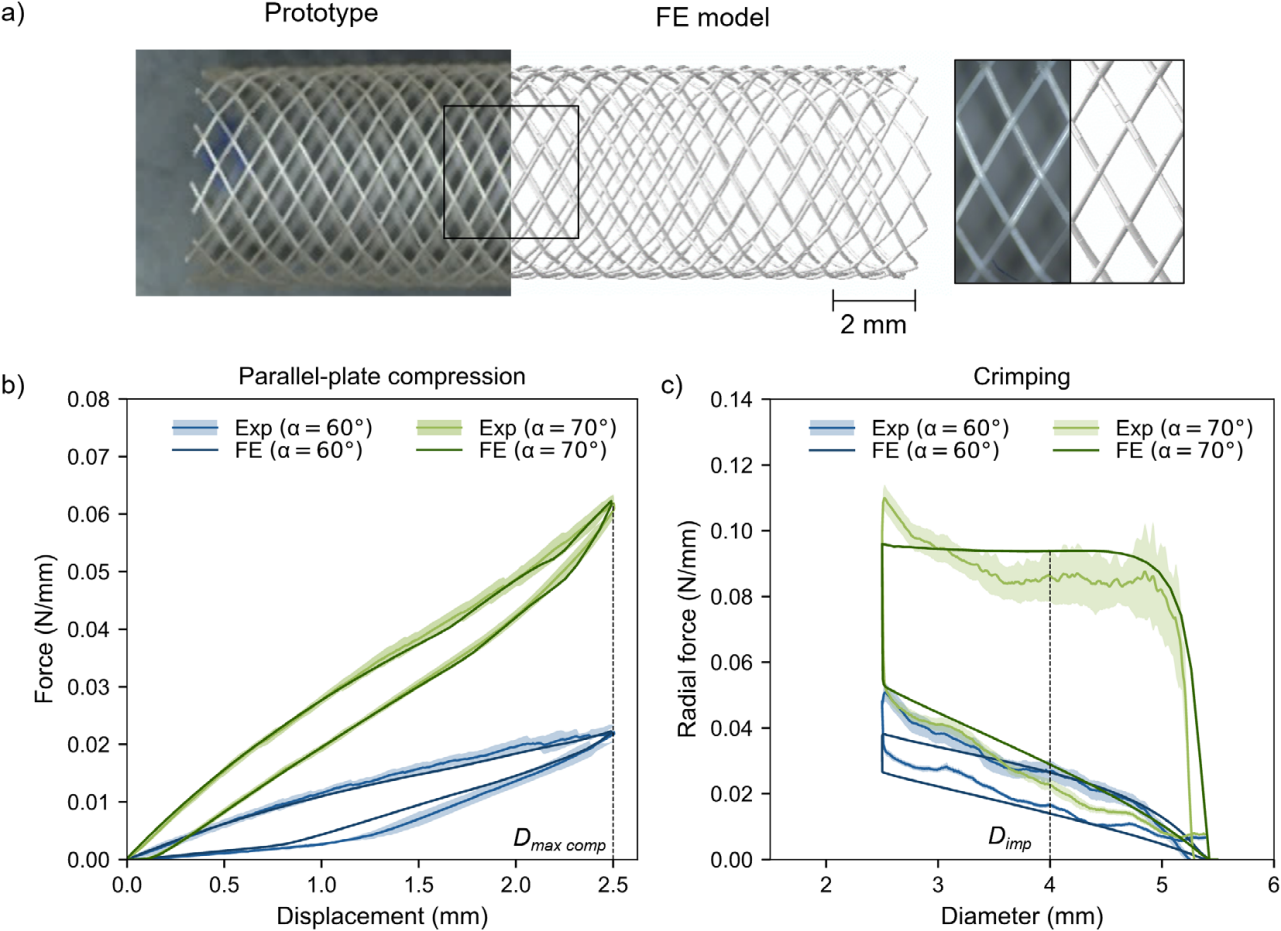
Validation of the FE model of the PLLA braided stent against experimental data from Lucchetti et al. [13]. (a) Visual comparison of the PLLA stent (α = 60°) prototype and FE model, (b, c) FE analysis results and experimental data of the PLLA stents (α = 60° and α = 70°) for (b) parallel‐plate compression and (c) crimping. Experimental data are represented in terms of average values and standard deviation, which is depicted as a shadow. Force and radial force values are divided by *l*_0_. *D*_*imp*_: implantation diameter; *D*_max comp_: diameter at maximum compression.

Figure 7a displays the FE model of the PLLA braided stent alongside the prototype used in the experimental study by Lucchetti et al. [13], demonstrating a good geometric correspondence. Figure 7b,c compares the forces computed by the FE model with the experimental measurements from Lucchetti et al. [13] for the parallel‐plate and crimping tests, respectively. A good agreement is observable between experimental and computational data for both the calibrated (α = 60°) and the validation (α = 70°) models for both bench tests. Specifically, for the parallel‐plate compression, the relative difference in the force value at maximum compression (Figure 7b) between the average experimental and the computational data was 1.0% for both the calibrated and the validation models. For the crimping test, relative differences in terms of radial resistive force (RRF) and COF at the implantation diameter *D*_imp_ = 4 mm (Figure 7c) between the average experimental and computational data were, respectively, 2% and 10% for the calibrated model and 9% and 35% for the validation model. Furthermore, the mean absolute relative error was calculated and found to be 20% and 5% for the calibrated and validation models of the parallel‐plate compression and 25% and 17% for the calibrated and validation models of the crimping.

**FIGURE 8:**
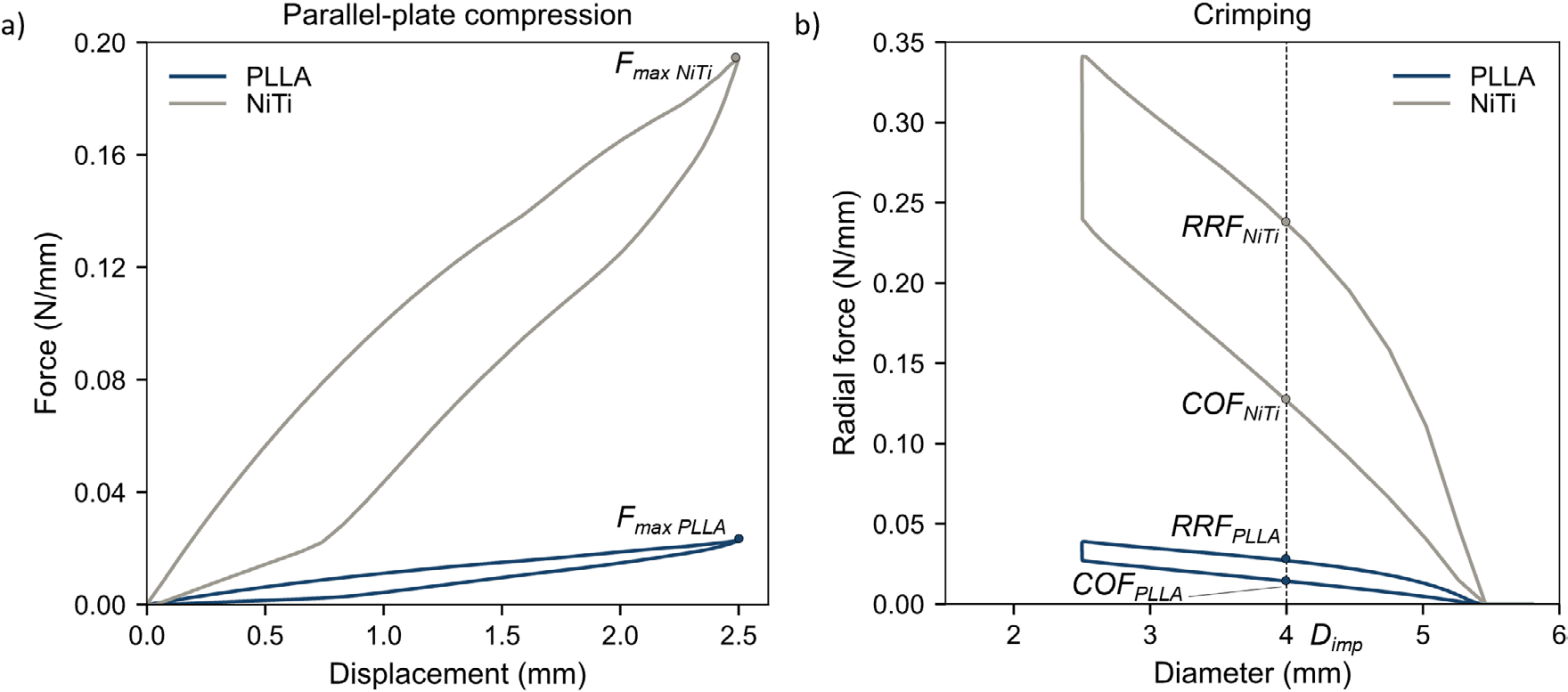
FE analysis of the PLLA and NiTi braided stents under (a) parallel‐plate compression and b) crimping. Force and radial force values were divided by *l*_0_. COF_*NiTi*_ and COF_*PLLA*_: chronic outward force at the implantation diameter *D*_imp_ for NiTi and PLLA stents, respectively. *F*_maxNiTi_ and *F*_maxPLLA_: force at maximum compression for NiTi and PLLA stents, respectively; RRF_*NiTi*_ and RRF_*PLLA*_: radial resistive force at the implantation diameter *D*_imp_ for NiTi and PLLA stents, respectively.

Figure 8 illustrates the results of the FE analysis of the PLLA and NiTi braided stents of both parallel‐plate compression (Figure 8a) and crimping (Figure 8b) tests. The values of *F*_max_, as well as RRF and COF measured at the implantation diameter *D*_imp_ = 4 mm, are reported in Table 4. Overall, the results highlight that forces generated by the PLLA stent were markedly lower than those produced by the NiTi device in both bench tests.

**TABLE 4:** Forces computed in the bench tests.

	*F*_max_ (N/mm)	RRF (N/mm)	COF (N/mm)
PLLA stent	0.023	0.025	0.013
NiTi stent	0.194	0.230	0.119

*Note:* The computed force values are represented in Figure 8.

Abbreviations: COF: chronic outward force; *F*_max_: force at maximum compression; RRF: radial resistive force.

### 3.2 Virtual Stent Implantation

#### 3.2.1 Verification of the FE Simulation

Table 5 presents the RIF magnitudes obtained from the stent implantation analysis, compared to the COF values computed in the crimping test, for the three investigated arterial wall conditions. The results demonstrate a good correspondence between the RIF and COF magnitudes for all arterial wall conditions, confirming the credibility of the FE analysis in calculating interaction forces during implantation. Minor discrepancies were attributed to the variable arterial diameter along its length, which differs from the fixed diameter used in the COF calculations.

**TABLE 5:** Comparison of RIF magnitudes, computed in the implantation analysis and COF values, computed in the crimping test, for the three arterial wall conditions.

	Arterial wall condition	Average diameter (mm)	RIF (N/mm)	COF (N/mm)
PLLA stent	Low stiffness	3.63	0.021	0.025
	Medium stiffness	3.64	0.021	0.025
	High stiffness	3.79	0.020	0.023
NiTi stent	Low stiffness	4.08	0.164	0.165
	Medium stiffness	3.86	0.171	0.188
	High stiffness	4.06	0.168	0.172

#### 3.2.2 Evaluation of the Treatment Performance

##### 3.2.2.1 Stent Stress State

**FIGURE 9:**
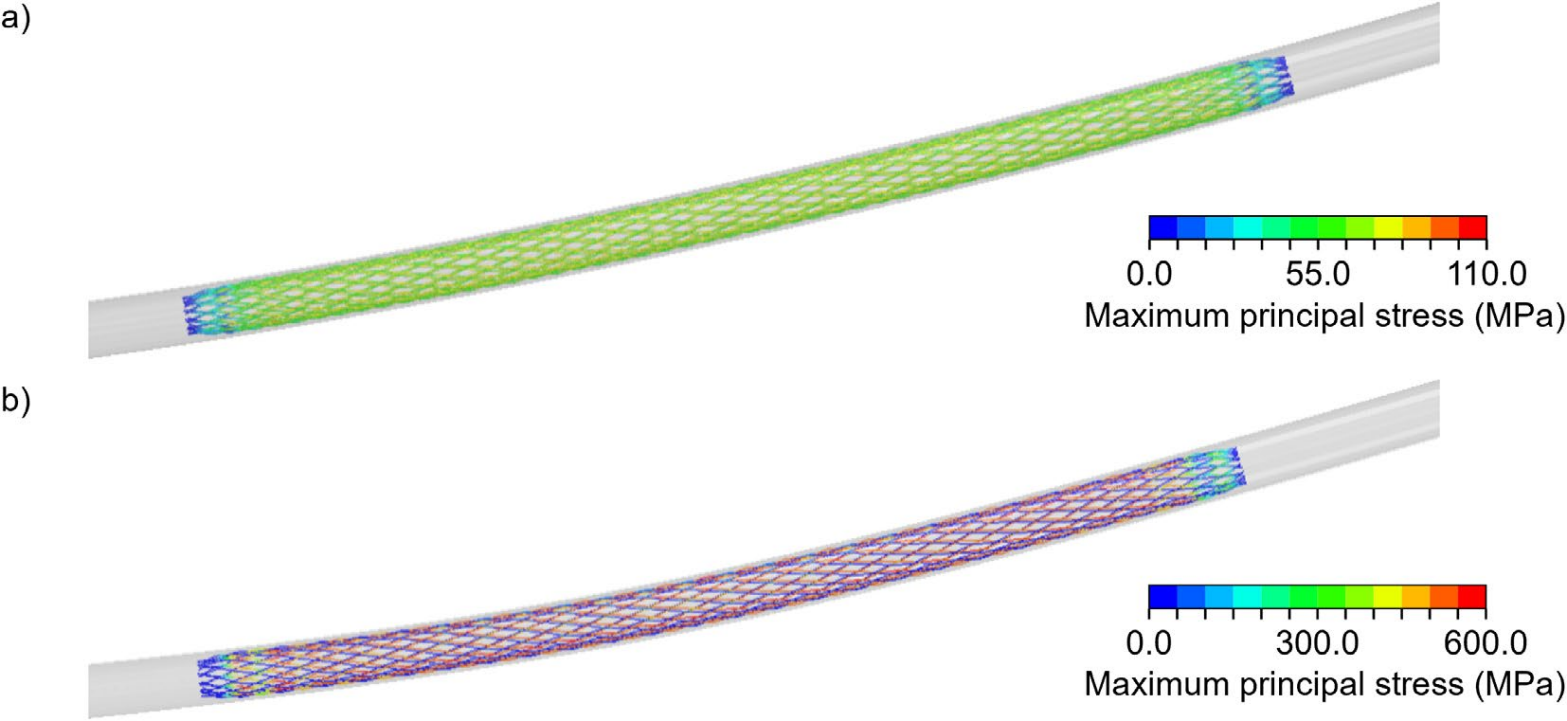
Maximum principal stress during insertion into the 6‐Fr catheter sheath of (a) PLLA and (b) NiTi stents.

Figure 9 illustrates the maximum principal stress of PLLA and NiTi braided stents when the device is inserted into the catheter sheath before the implantation (Figure 5e). The peak values of the maximum principal stress were 87 and 582 MPa for the PLLA and NiTi devices, respectively. For the PLLA stent, the peak stress value was below the yield stress values of the material (Table 1), indicating the absence of permanent deformations during the stent implantation. For the NiTi stent, the peak stress value was below the ultimate tensile strength value of the material, conservatively considering a value of 973 MPa based on tensile tests on NiTi wires [31].

##### 3.2.2.2 LG

**FIGURE 10:**
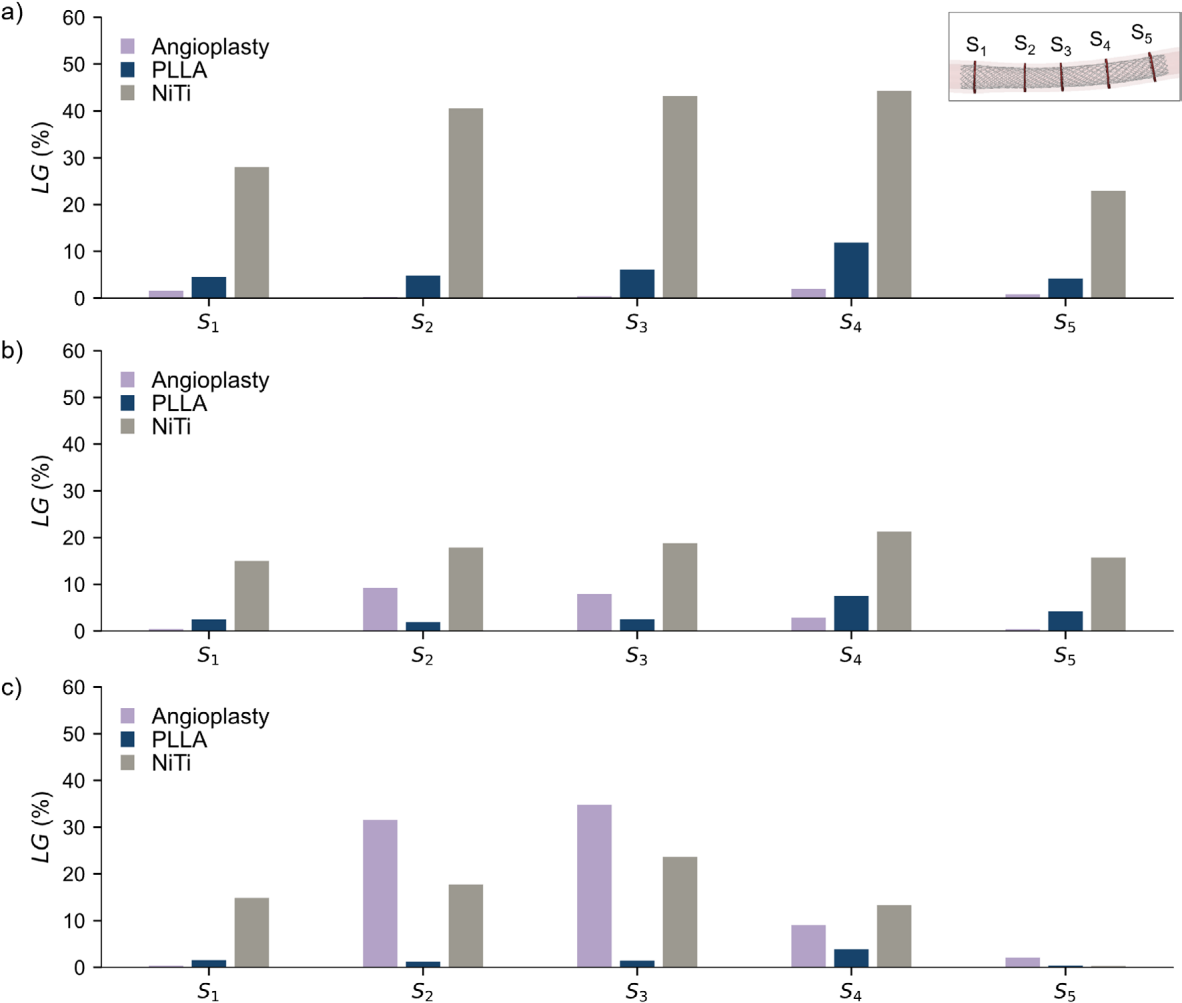
LG induced by the angioplasty and stent implantation for (a) low, (b) medium and (c) high stiffness arterial wall conditions, computed at the cross‐sections *S*_1_–*S*_5_. The LG induced by stent implantation was computed considering the artery's post‐angioplasty configuration as initial reference.

Figure 10 presents the LG values for the three arterial wall conditions following both angioplasty and implantation of both PLLA and NiTi stents. It can be observed that globally the LG induced by angioplasty increases as vessel stiffness increases. Specifically, at cross‐sections *S*_1_–*S*_3_, the LG increases from 0.3%, 0.2% and 1.8% to 31.3%, 34.5% and 8.8%, going from low to high stiffness arterial wall conditions. Regarding stent implantation, the PLLA stent induced overall markedly lower LG compared to the NiTi device. In particular, the former induced a maximum LG in *S*_4_ of 11.6%, 7.3% and 3.6%, whereas the latter generated LG in *S*_4_ of 44%, 21% and 13% under low, medium and high stiffness arterial wall conditions, respectively.

##### 3.2.2.3 ISA

**FIGURE 11:**
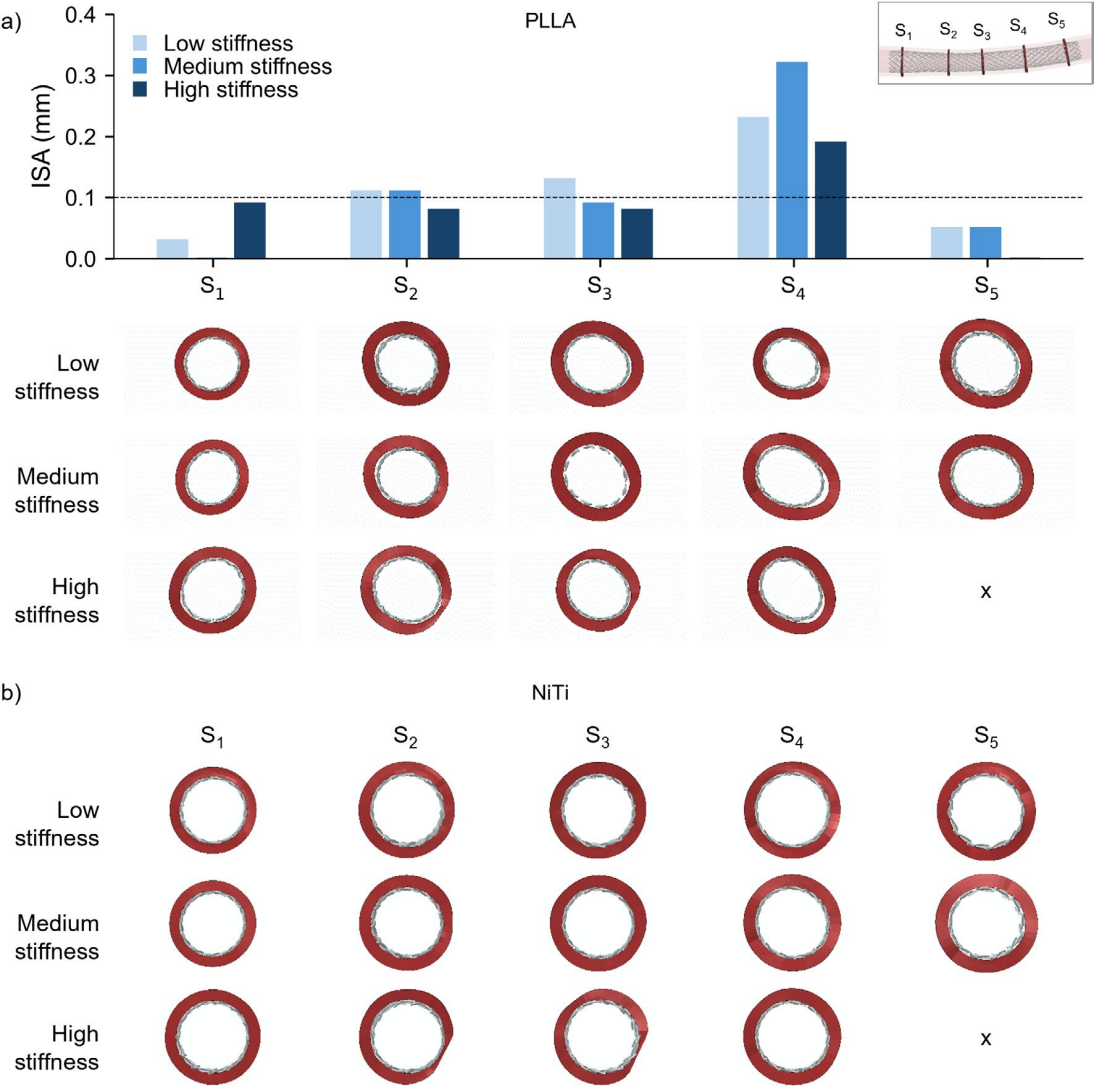
ISA of the implanted (a) PLLA and (b) NiTi braided stents for low‐, medium‐ and high‐stiffness arterial wall conditions. The critical ISA threshold is marked by the black dashed line. The NiTi stent exhibited ISA values equal to zero under all three arterial wall conditions and in all cross‐sections, hence its bar plot is not included. *Note:* For the implantation under high stiffness arterial wall condition, both stents were absent in slice *S*_5_ due the effect of angioplasty and stent foreshortening (denoted by X).

Figure 11 presents the ISA values resulting from the implantation of the PLLA and NiTi braided stents for the three arterial wall conditions, including cross‐sectional views of the artery (i.e., *S*_1_–*S*_5_).

Figure 11a shows that the PLLA device exhibited malapposition in all five cross‐sections, with ISA values greater than zero. The maximum ISA values occurred at *S*_4_ for all three arterial wall conditions, where the artery section assumes an elliptical shape, due to the presence of non‐eccentric plaques. Additionally, at cross‐sections *S*_2_, *S*_3_ and *S*_4_, the PLLA stent displayed ISA values exceeding the critical threshold for malapposition [30]. In contrast, the NiTi stent exhibited ISA values equal to zero under all three arterial wall conditions and in all cross‐sections, with no visible malapposition (Figure 11b).

##### 3.2.2.4 Contact Pressure

**FIGURE 12:**
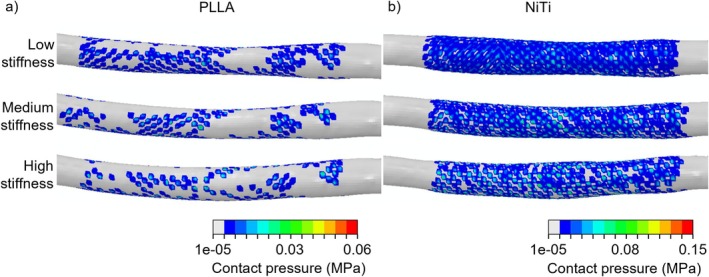
Contact pressure exerted on the artery by the (a) PLLA and (b) NiTi braided stents, for low‐, medium‐ and high‐stiffness arterial wall condition.

Figure 12 illustrates the contact pressure on the artery following the implantation of the PLLA and NiTi stents, considering the three arterial wall conditions. The PLLA stent generated lower contact pressures compared to the NiTi device across all arterial wall conditions, attributed to the lower forces generated by the PLLA stent (Table 5). Additionally, the PLLA stent exhibited a non‐uniform pressure distribution along the artery and regions of malapposition. In contrast, the NiTi stent showed a uniform distribution of the contact pressure along the artery and the absence of malapposition.

## 4 Discussion

In this study, the mechanical properties and short‐term implantation performance of a PLLA braided stent in the SFA were evaluated through FE analysis and compared with that of a NiTi device having identical geometrical features. The results from the in silico bench tests demonstrated the self‐expanding capability of the PLLA stent despite its lower mechanical performance compared to its NiTi counterpart. However, the study highlighted the limited success of the short‐term implantation performance of the evaluated PLLA stent, which demonstrated a higher risk of undergoing permanent deformations during the procedure, limited LG and regions of malapposition. In contrast, NiTi devices showed a lower risk of permanent deformations, induced a higher LG and resulted in no malapposition. The results emphasise the need for further material and device optimisation to improve the performance of PLLA braided stents. Nevertheless, the potential application of PLLA stents for the intravascular treatment of other pathologies not characterised by severe stenosis and vessel eccentricity, such as in neurovascular interventions as flow diverters, remains a possibility.

Bioresorbable braided stents are a promising alternative to permanent devices for treating SFAs, offering advantages in terms of reduced late‐stage inflammation, minimised imaging artifacts, fewer complications during secondary surgeries and a reduced need for long‐term antiplatelet therapy [10, 11, 12]. Prior research has focussed only on the mechanical characterisation of PLLA braided stents [13, 14, 15, 16, 17], demonstrating their self‐expanding capabilities, high flexibility and low radial stiffness compared to metallic devices. Since, to the best of the authors' knowledge, no study in the literature has yet investigated the short‐term implantation performance of PLLA braided stents in SFAs, the present study aimed to address this gap. To this end, a computational framework was implemented which enabled the virtual mechanical characterisation of the stents in two bench tests and the virtual implantation of braided devices in the SFA, accounting for different arterial conditions.

Regarding the models' accuracy, the two simulated bench tests were shown to closely match the experimental data published by Lucchetti et al. [13]. A slightly lower accuracy was observed in the crimping test compared to the parallel‐plate compression test, which may be attributable to the greater filament sliding and the globally higher stress levels observed during the radial crimping test. Moreover, good agreement was achieved between the RIF and COF values, confirming the calculation accuracy of the interaction forces during stent implantation. The in silico mechanical characterisation of the PLLA stent highlighted a good self‐expandability of the polymeric device despite the lower forces generated compared to the NiTi stent with identical geometrical characteristics. The analysis of stent implantation under different arterial wall conditions revealed the limited performance of the PLLA stent evaluated compared to its NiTi counterpart. The peak stress values arising during stent insertion into the catheter remained below the materials' yield limits for both the PLLA and NiTi devices. However, for the PLLA stent, the registered values approached this limit, thus providing a small safety margin and highlighting a potential risk of permanent deformations during the actual implantation procedure. This small safety margin may be critical during implantation in tortuous vascular anatomies, where catheter bending during navigation could further increase the loading of the stent. Regarding the stent short‐term implantation performance, results showed that, for both PLLA and NiTi stents, different outcomes were related to the different arterial wall conditions (i.e., low, medium or high stiffness). More specifically, the LG induced by angioplasty increased with increasing vessel stiffness, as higher stresses originated in the artery, reaching a range of 9%–34% under the most rigid condition. In contrast, in less stiff arteries, the dominant effect of the elastic recoil led to lower LG values, ranging from 0% to 1%. This in turn affected the stent impact on inducing further LG. For the PLLA stent, the observed LG ranged from 4% to 11% in the least rigid artery to 1%–3% in the most rigid condition. In contrast, the NiTi stent achieved higher LG of 23%–44% and 13%–23% in the least and most rigid conditions, respectively. The effect of the low COF of the PLLA stent was evident not only in the lower LG compared to the NiTi device, but also in high values of ISA and non‐uniform distribution of contact pressure on the arterial wall. Specifically, ISA values exceeding the critical threshold, along with areas of zero pressure on the arterial wall, were primarily observed in regions where the artery's cross‐section assumed an elliptical shape. Such pronounced malapposition may be associated with an increased risk of delayed stent coverage, persistent malapposition at follow‐ups and an increased risk of stent thrombosis due to induced fluid dynamic disturbances [26, 30]. In contrast, the NiTi stent exhibited no ISA and induced a uniform distribution of pressure on the arterial wall, indicating a high adherence between the stent and the artery. However, it is important to note that high LG values induced by the NiTi stent may potentially lead to arterial tissue damage.

Although the study highlights open challenges still associated with polymeric stents when implanted in an SFA, the authors do not exclude potential future applications in other settings that are not characterised by severe stenosis and vessel eccentricity, such as in neurovascular intervention as flow diverters. Moreover, the study emphasises the need for further efforts to enhance the mechanical properties of these devices. From the material viewpoint, spinning process parameters should be investigated to improve the orientation of molecular chains in the filament and increase crystallinity, thereby enhancing the material's mechanical properties [32]. From a device perspective, optimising parameters such as braiding angle, number of filaments and filament diameter can enhance the mechanical properties of PLLA braided stents and, consequently, improve treatment performance. Nevertheless, the optimization study conducted by Carbonaro et al. [16] showed that even after geometric optimization, the resulting COF remains markedly inferior to that of a conventional NiTi braided stent (see Table 4). In this regard, a promising approach that should be further investigated may involve moving away from conventional braiding patterns and exploring alternative braiding designs, as previously explored in Lucchetti et al. [13].

The study presents some limitations and modelling assumptions. First, since only CT scan data were available, the artery geometry was reconstructed assuming a constant thickness and a homogeneous and isotropic material, following the approach proposed by Noble et al. [27], without distinguishing between calcified and healthy tissue. Moreover, plaque fracture was modelled by considering perfect plasticity, assuming that after yielding the material has no resistance. Additionally, no post‐intervention imaging was available, as the stents were not implanted in vivo. As a result, the implanted stent configuration could not be validated using in vivo post‐implantation imaging. Nevertheless, the credibility of the FE simulation of stent implantation was successfully verified by comparing the RIF exerted by the virtually deployed stent with the COF measured in the crimping test. The geometry of the SFA from a single patient was considered, in conjunction with the comparison of a single geometry of a PLLA and NiTi braided stent. In this regard, further investigations could be conducted on SFAs with different anatomies and degrees of stenosis. However, due to the inherently low mechanical properties of the PLLA stent, a low treatment performance is expected for the PLLA stent across different SFA anatomies and pathological conditions. In addition, while the present study focuses on the comparison between a PLLA and NiTi stent, further investigations should be conducted on the implantation analysis of PLLA braided stents with different geometrical parameters and braiding patterns. The mechanical properties of NiTi were based on a study by McKenna et al. [4]. However, NiTi wire properties can vary substantially depending on the specific chemical composition and processing of the material [31]. Moreover, experimental data on friction coefficients between NiTi wires were not available and were assumed to be equal to those of the PLLA device. Despite these limitations, the computational procedure effectively compared the mechanical characteristics and short‐term implantation performance of PLLA and NiTi braided stents and could be extended to other vascular stents with different geometries and materials.

## 5 Conclusions

A computational procedure was developed to compare the mechanical characteristics and short‐term performance of a PLLA and a NiTi braided stent for the treatment of lower limb arteries. The FE models of both parallel‐plate compression and crimping tests of the PLLA stent were successfully validated against experimental data. The comparison of the mechanical characteristics evaluated in the two bench tests revealed a substantial difference between the two devices, with the PLLA stent generating much lower force magnitudes. The FE simulation of stent implantation highlighted the limited short‐term treatment performance of the PLLA stent evaluated in terms of risk for permanent deformations, low LG, high values of ISA and a non‐uniform distribution of contact pressure on the arterial wall. Finally, the developed procedure enabled virtual investigation of the biomechanical interaction between the stent and the artery and could be extended to evaluate the treatment performance of other vascular stents.

## Author Contributions

**Agnese Lucchetti:** conceptualization, methodology, software, validation, investigation, data curation, visualisation, writing – original draft, writing – review and editing. **Levi G. Juhl:** methodology, software, investigation, data curation, visualisation, writing – review and editing. **Anna Corti:** methodology, data curation, writing – review and editing. **Alissa Zaccaria:** methodology, writing – review and editing. **Thomas Gries:** funding acquisition, writing – review and editing. **Claudio Chiastra:** methodology, writing – review and editing. **Ted J. Vaughan:** methodology, supervision, funding acquisition, writing – review and editing. **Dario Carbonaro:** conceptualization, methodology, investigation, supervision, writing – original draft, writing – review and editing.

## Ethics Statement

The authors have nothing to report.

## Consent

The authors have nothing to report.

## Conflicts of Interest

The authors declare no conflicts of interest.

## Supporting information


**Data S1:** Supporting Information.

## Data Availability

The data that support the findings of this study are available from the corresponding author upon reasonable request.

## References

[1] C. Pan , Y. Han , and J. Lu , “Structural Design of Vascular Stents: A Review,” Micromachines 12, no. 7 (2021), 10.3390/mi12070770. PMC8305143 34210099

[2] M. Schillinger and E. Minar , “Percutaneous Treatment of Peripheral Artery Disease: Novel Techniques,” Circulation 126, no. 20 (2012), 10.1161/CIRCULATIONAHA.111.036574. 23147770

[3] J. N. MacTaggart , N. Y. Phillips , C. S. Lomneth , et al., “Three‐Dimensional Bending, Torsion and Axial Compression of the Femoropopliteal Artery During Limb Flexion,” Journal of Biomechanics 47, no. 10 (2014), 10.1016/j.jbiomech.2014.04.053. 24856888

[4] C. G. McKenna and T. J. Vaughan , “A Finite Element Investigation on Design Parameters of Bare and Polymer‐Covered Self‐Expanding Wire Braided Stents,” Journal of the Mechanical Behavior of Biomedical Materials 115 (2021): 104305, 10.1016/j.jmbbm.2020.104305. 33454463

[5] J. H. Kim , T. J. Kang , and W.‐R. Yu , “Mechanical Modeling of Self‐Expandable Stent Fabricated Using Braiding Technology,” Journal of Biomechanics 41, no. 15 (2008): 3202–3212, 10.1016/j.jbiomech.2008.08.005. 18804764

[6] Q. Zheng , H. Mozafari , Z. Li , et al., “Mechanical Characterization of Braided Self‐Expanding Stents: Impact of Design Parameters,” Journal of Mechanics in Medicine and Biology 19, no. 06 (2019): 1950038, 10.1142/S0219519419500386.

[7] K. Bishu and E. J. Armstrong , “Supera Self‐Expanding Stents for Endovascular Treatment of Femoropopliteal Disease: A Review of the Clinical Evidence,” Vascular Health and Risk Management 11 (2015): 387–395, 10.2147/VHRM.S70229. 26203255 PMC4508067

[8] S. Vanaei , M. Hashemi , A. Solouk , et al., “Manufacturing Processing and Characterization of Self‐Expanding Metallic Stents A Comprehensive Review,” Bioengineering (Basel, Switzerland) 11, no. 10 (2024), 10.3390/bioengineering11100983. PMC11505524 39451359

[9] A. Zaccaria , F. Migliavacca , G. Pennati , and L. Petrini , “Modeling of Braided Stents: Comparison of Geometry Reconstruction and Contact Strategies,” Journal of Biomechanics 107 (2020): 109841, https://doi.org/10.1016/j.jbiomech.2020.109841. 32517859 10.1016/j.jbiomech.2020.109841

[10] H. Y. Ang , Y. Y. Huang , S. T. Lim , P. Wong , M. Joner , and N. Foin , “Mechanical Behavior of Polymer‐Based vs. Metallic‐Based Bioresorbable Stents,” Journal of Thoracic Disease 9 (2017): S923–S934, 10.21037/jtd.2017.06.30. 28894598 PMC5583085

[11] B. Forrestal , B. C. Case , C. Yerasi , A. Musallam , C. Chezar‐Azerrad , and R. Waksman , “Bioresorbable Scaffolds: Current Technology and Future Perspectives,” Rambam Maimonides Medical Journal 11, no. 2 (2020): e0016, 10.5041/RMMJ.10402. 32374257 PMC7202443

[12] H. Jinnouchi , S. Torii , A. Sakamoto , F. D. Kolodgie , R. Virmani , and A. V. Finn , “Fully Bioresorbable Vascular Scaffolds: Lessons Learned and Future Directions,” Nature Reviews. Cardiology 16, no. 5 (2019): 286–304, 10.1038/s41569-018-0124-7. 30546115

[13] A. Lucchetti , C. Emonts , A. Idrissi , T. Gries , and T. J. Vaughan , “An Experimental Investigation of the Mechanical Performance of PLLA Wire‐Braided Stents,” Journal of the Mechanical Behavior of Biomedical Materials 138 (2023): 105568. 36459705 10.1016/j.jmbbm.2022.105568

[14] A. Lucchetti , F. Caronna , L. Rocher , et al., “Investigation of the Degradation Behaviour of Poly‐L‐Lactic Acid Braided Stents Under Real‐Time and Accelerated Conditions,” Polymer Testing 141 (2024): 108632, 10.1016/j.polymertesting.2024.108632.

[15] G. Zhao , M. Liu , D. Deng , et al., “Effects of Constraint Between Filaments on the Radial Compression Properties of Poly (l‐Lactic Acid) Self‐Expandable Braided Stents,” Polymer Testing 93 (2021): 106963, 10.1016/j.polymertesting.2020.106963.

[16] D. Carbonaro , A. Lucchetti , A. L. Audenino , T. Gries , T. J. Vaughan , and C. Chiastra , “Multi‐Objective Design Optimization of Bioresorbable Braided Stents,” Computer Methods and Programs in Biomedicine 242 (2023): 107781, 10.1016/j.cmpb.2023.107781. 37683458

[17] J. Li , J. Cheng , X. Hu , et al., “A Hazardous Boundary of Poly(L‐Lactic Acid) Braided Stent Design: Limited Elastic Deformability of Polymer Materials,” Journal of the Mechanical Behavior of Biomedical Materials 138 (2023): 105628, 10.1016/j.jmbbm.2022.105628. 36543082

[18] A. Lucchetti , “Experimental and Model‐Based Development of Bioresorbable Braided Stents” (RWTH Aachen University, 2024), 10.18154/RWTH-2024-06968.

[19] F. Auricchio and R. L. Taylor , “Shape‐Memory Alloys: Modelling and Numerical Simulations of the Finite‐Strain Superelastic Behavior,” Computer Methods in Applied Mechanics and Engineering 143, no. 1–2 (1997): 175–194, 10.1016/S0045-7825(96)01147-4.

[20] ISO , ISO 25539–2:2020: Cardiovascular Implants—Endovascular Devices. Part 2: Vascular Stents (ISO, 2020).

[21] L. Antonini , L. Mandelli , F. Berti , G. Pennati , and L. Petrini , “Validation of the Computational Model of a Coronary Stent: A Fundamental Step Towards In Silico Trials,” Journal of the Mechanical Behavior of Biomedical Materials 122 (2021): 104644, 10.1016/j.jmbbm.2021.104644. 34186285

[22] A. Zaccaria , F. Migliavacca , D. Contassot , et al., “Finite Element Simulations of the ID Venous System to Treat Venous Compression Disorders: From Model Validation to Realistic Implant Prediction,” Annals of Biomedical Engineering 49, no. 6 (2021): 1493–1506, 10.1007/s10439-020-02694-8. 33398616 PMC8137589

[23] S. Giannopoulos , E. A. Secemsky , P. A. Schneider , and E. J. Armstrong , “Concomitant Drug‐Coated Balloon Angioplasty With Bail‐Out Use of Eluvia Drug‐Eluting Stent: Is There Any Downside to a Double Dose of Paclitaxel?,” Journal of Invasive Cardiology 34, no. 6 (2022): E469–E476, 10.25270/jic/21.00354. 35652710 PMC12856877

[24] I. Nazari , S. M. Mousavi , A. Sadeghpour , S. M. Alamshah , and M. Dastoorpoor , “Comparison of Effectiveness of Drug‐Coated Balloon Angioplasty Versus Plain Balloon Angioplasty in Chronic Lower Extremity Ischemia Patients,” International Journal of General Medicine 13 (2020): 609–615, 10.2147/IJGM.S256240. 32982378 PMC7501961

[25] M. Jadidi , S. A. Razian , E. Anttila , et al., “Comparison of Morphometric, Structural, Mechanical, and Physiologic Characteristics of Human Superficial Femoral and Popliteal Arteries,” Acta Biomaterialia 121 (2021): 431–443, 10.1016/j.actbio.2020.11.025. 33227490 PMC7855696

[26] M. Bernini , R. Hellmuth , M. O'Sullivan , et al., “Shape‐Setting of Self‐Expanding Nickel‐Titanium Laser‐Cut and Wire‐Braided Stents to Introduce a Helical Ridge,” Cardiovascular Engineering and Technology 15 (2024): 317–332, 10.1007/s13239-024-00717-2. 38315312 PMC11239776

[27] C. Noble , K. D. Carlson , E. Neumann , et al., “Patient Specific Characterization of Artery and Plaque Material Properties in Peripheral Artery Disease,” Journal of the Mechanical Behavior of Biomedical Materials 101 (2020): 103453, 10.1016/j.jmbbm.2019.103453. 31585351 PMC6889048

[28] E. M. Cunnane , J. J. E. Mulvihill , H. E. Barrett , et al., “Mechanical, Biological and Structural Characterization of Human Atherosclerotic Femoral Plaque Tissue,” Acta Biomaterialia 11 (2015): 295–303, 10.1016/j.actbio.2014.09.024. 25242646

[29] A. Corti , M. Colombo , F. Migliavacca , et al., “Multiscale Agent‐Based Modeling of Restenosis After Percutaneous Transluminal Angioplasty: Effects of Tissue Damage and Hemodynamics on Cellular Activity,” Computers in Biology and Medicine 147 (2022): 105753, 10.1016/j.compbiomed.2022.105753. 35797890

[30] F. Prati , G. Guagliumi , G. S. Mintz , et al., “Expert Review Document Part 2: Methodology, Terminology and Clinical Applications of Optical Coherence Tomography for the Assessment of Interventional Procedures,” European Heart Journal 33, no. 20 (2012): 2513–2520, 10.1093/eurheartj/ehs095. 22653335 PMC3470836

[31] D. Carbonaro , E. Villa , D. Gallo , U. Morbiducci , A. L. Audenino , and C. Chiastra , “Designing the Mechanical Behavior of NiTi Self‐Expandable Vascular Stents by Tuning the Heat Treatment Parameters,” Journal of the Mechanical Behavior of Biomedical Materials 158 (2024): 106653, 10.1016/j.jmbbm.2024.106653. 39074439

[32] Y. Nishimura , A. Takasu , Y. Inai , and T. Hirabayashi , “Melt Spinning of Poly(L‐Lactic Acid) and Its Biodegradability,” Journal of Applied Polymer Science 97, no. 5 (2005): 2118–2124, 10.1002/app.21915.

