# Supplementary material for: From Bench Testing to Virtual Implantation: A Comparative Study Between Poly‐l‐Lactic Acid and Nickel‐Titanium Braided Stents
Source: Int J Numer Method Biomed Eng. 2025 Aug 7;41(8):e70078. doi: 10.1002/cnm.70078 (PMC12331417; doi:10.1002/cnm.70078)
Supplement: Supplementary file 1 — Data S1: Supporting Information. [file CNM-41-e70078-s001.docx]

**From bench testing to virtual implantation: A comparative study between poly-L-lactic acid and nickel-titanium braided stents**

Agnese Lucchetti^1*^, Levi G. Juhl^1^, Anna Corti^2^, Alissa Zaccaria^3^, Thomas Gries^1^,

Claudio Chiastra^4^, Ted J. Vaughan^5#^, Dario Carbonaro^4#^

# Ted J. Vaughan and Dario Carbonaro contributed equally to this work and are co-last authors.

1. Institut für Textiltechnik of RWTH Aachen University, Aachen, Germany

2. Department of Electronics, Information and Bioengineering, Politecnico di Milano, Milan, Italy

3. Consorzio Intellimech, Bergamo, Italy

4. Polito^BIO^Med Lab, Department of Mechanical and Aerospace Engineering, Politecnico di Torino, Turin, Italy

5. Biomechanics Research Centre (BioMEC), School of Engineering and Institute for Health Discovery and Innovation, College of Science and Engineering, University of Galway, Galway, Ireland

***Corresponding Author:**

Name: Dr.-Ing. Agnese Lucchetti

Affiliation: Institut für Textiltechnik of RWTH Aachen University, Aachen, Germany

Current address: Department of Cardiovascular Engineering, Institute of Applied Medical Engineering, Medical Faculty, RWTH Aachen University, Forckenbeckstraße 55, 52074, Aachen, Germany

Email address: [lucchetti@ame.rwth-aachen.de](mailto:lucchetti@ame.rwth-aachen.de)

# Supplementary Material

## Balloon FE model

The angioplasty balloon was modelled following the approach by Corti et al. [(2022)](#_CTVL0012b0c31bb5348469c8f544ba5915ecbbf), replicating the commercially available Armada 35 Balloon Dilation Catheter (Abbott, Illinois, USA). The size of the balloon was chosen based on the lesion length and artery diameter. Specifically, for a lesion measuring approximately 30 mm, the balloon size with a length of 40 mm and a diameter of 4 mm was chosen from the manufacturer datasheet.

The balloon geometry was created in SolidWorks (Dassault Systèmes SolidWorks Corporation, Waltham, MA, USA) and consisted of 30 wings (see Fig S1), which unfold when the balloon is pressurized and expanded [(Chiastra et al., 2015](#_CTVL001aa0ffc096bcb471f80a5728858e3901f)[; Chiastra et al., 2018)](#_CTVL0013f847d6d2a2b40268184b4d79fc05c18). The geometry was created in its crimped state with an external diameter of the balloon main body of 1.75 mm. The tips of the balloon represent the connection to the catheter and were characterized by a diameter and a length of 1.67 mm and 4.6 mm, respectively (see Fig S1). The balloon nominal diameter, defined as the diameter of the fully unfolded, but not yet pressurized balloon, was determined from the manufacturer’s compliance chart. More specifically, data for a balloon with inflation diameters from 4 mm to 4.5 mm were linearly interpolated, and the diameter at 0 MPa (unfolded, unpressurized state) was extrapolated. This resulted in a balloon nominal diameter of 3.72 mm.

Fig S1: CAD model of the multi-wing angioplasty balloon in its crimped state.

The balloon geometry was discretized with 15,000 M3D4R elements with a thickness of 0.025 mm [(Chiastra et al., 2015)](#_CTVL001aa0ffc096bcb471f80a5728858e3901f). A linear-elastic isotropic material model was adopted, with an elastic modulus of 1.7 GPa, a Poisson’s ratio of 0.45, and a density of 1 g/cm3 [(Corti et al., 2022)](#_CTVL0012b0c31bb5348469c8f544ba5915ecbbf). Notably, the elastic modulus was determined through a calibration procedure by performing free expansion simulations to fit the diameter vs. pressure curve provided by the manufacturer.

## Delivery system FE model

The delivery system was modelled following the approach of Zaccaria et al. [(2021)](#_CTVL0018832a37168f54f34b194598b67a3d5d9) and comprised a nickel-titanium (NiTi) guidewire, a Polytetrafluoroethylene catheter sheath and anti-jump system, as represented in Fig S2. Specifically, the anti-jump system prevents the stent from moving while the catheter sheath is withdrawn during the deployment procedure. Each part was created and meshed in Abaqus/Explicit (Dassault Systèmes Simulia Corp., Johnston, RI, USA). The guidewire was meshed with B31 beam elements, with a diameter of 0.35 mm. The catheter sheath and anti-jump system were meshed with S4R quadrilateral shell elements, with a thickness of 0.11 mm. As in Zaccaria et al. [(2021)](#_CTVL0018832a37168f54f34b194598b67a3d5d9), both NiTi and PTFE materials were modelled as linear-elastic with material parameters taken from the same authors. These are summarised in Table S1.

Fig S2: FE model of the delivery system composed by the catheter sheath, anti-jump system and guidewire.

Table S1: Material parameters of the delivery system components [(Zaccaria et al., 2021)](#_CTVL0018832a37168f54f34b194598b67a3d5d9).

| Component | Material | Young’s modulus | Poisson’s ratio | Density |
| --- | --- | --- | --- | --- |
|  |  | (GPa) | (-) | (g/cm^3^) |
| Guidewire | NiTi | 50 | 0.3 | 6.45 |
| Catheter sheath | PTFE | 0.45 | 0.45 | 2.2 |
| Anti-jump system | PTFE | 0.45 | 0.45 | 2.2 |

References

Chiastra C, Grundeken MJ, Collet C, Wu W, Wykrzykowska JJ, Pennati G, et al. Biomechanical Impact of Wrong Positioning of a Dedicated Stent for Coronary Bifurcations: A Virtual Bench Testing Study. Cardiovasc Eng Tech 2018;9(3):415–26. https://doi.org10.1007/s13239-018-0359-9.

Chiastra C, Grundeken MJ, Wu W, Serruys PW, Winter RJ de, Dubini G, et al. First report on free expansion simulations of a dedicated bifurcation stent mounted on a stepped balloon. EuroIntervention 2015;10(11):e1-3. https://doi.org10.4244/EIJV10I11A226.

Corti A, Colombo M, Migliavacca F, Berceli SA, Casarin S, Rodriguez Matas JF, et al. Multiscale agent-based modeling of restenosis after percutaneous transluminal angioplasty: Effects of tissue damage and hemodynamics on cellular activity. Computers in biology and medicine 2022;147:105753. https://doi.org10.1016/j.compbiomed.2022.105753.

Zaccaria A, Migliavacca F, Contassot D, Heim F, Chakfe N, Pennati G, et al. Finite Element Simulations of the ID Venous System to Treat Venous Compression Disorders: From Model Validation to Realistic Implant Prediction. Annals of biomedical engineering 2021;49(6):1493–506. https://doi.org10.1007/s10439-020-02694-8.
